# Surveillance of Wild Birds for Avian Influenza Virus

**DOI:** 10.3201/eid1612.100589

**Published:** 2010-12

**Authors:** Bethany J. Hoye, Vincent J. Munster, Hiroshi Nishiura, Marcel Klaassen, Ron A.M. Fouchier

**Affiliations:** Author affiliations: Netherlands Institute for Ecology, Nieuwersluis, the Netherlands (B.J. Hoye, M. Klaassen);; Erasmus Medical Centre, Rotterdam, the Netherlands (V.J. Munster, R.A.M. Fouchier);; National Institute of Health, Hamilton, Montana, USA (V.J. Munster);; University of Utrecht, Utrecht, the Netherlands (H. Nishiura);; Japan Science and Technology Agency, Saitama, Japan (H. Nishiura);; Deakin University, Waurn Ponds, Victoria, Australia (M. Klaassen)

**Keywords:** Survey methodology, influenza A, disease reservoirs, wildlife, wild birds, waterfowl, infection, prevalence, viruses, perspective, *Suggested citation for this article*: Hoye BJ, Munster VJ, Nishiura H, Klaassen M, Fouchier RAM. Surveillance of wild birds for avian influenza virus. Emerg Infect Dis [serial on the Internet]. 2010 Dec [*date cited*]. http://dx.doi.org/10.3201/eid1612.100589

## Abstract

Recent demand for increased understanding of avian influenza virus in its natural hosts, together with the development of high-throughput diagnostics, has heralded a new era in wildlife disease surveillance. However, survey design, sampling, and interpretation in the context of host populations still present major challenges. We critically reviewed current surveillance to distill a series of considerations pertinent to avian influenza virus surveillance in wild birds, including consideration of what, when, where, and how many to sample in the context of survey objectives. Recognizing that wildlife disease surveillance is logistically and financially constrained, we discuss pragmatic alternatives for achieving probability-based sampling schemes that capture this host–pathogen system. We recommend hypothesis-driven surveillance through standardized, local surveys that are, in turn, strategically compiled over broad geographic areas. Rethinking the use of existing surveillance infrastructure can thereby greatly enhance our global understanding of avian influenza and other zoonotic diseases.

## Abstract 2

TOC Summary: A targeted, hypothesis-based approach and local surveys over broad geographic areas are needed.

Avian influenza virus (AIV) gained a high profile after the unprecedented bird-to-human transmission of highly pathogenic AIV (HPAIV) subtype H5N1 in 1997. Originating in Asia, HPAIV (H5N1) subsequently caused widespread deaths among wild and domestic birds in Southeast Asia and westward throughout Europe and Africa in 2005 and 2006. After ≈50 years of research in wild birds, a wide range of low-pathogenicity AIV (LPAIV) subtypes is known to circulate in numerous species (1,2–5), and LPAIVs are believed to perpetuate in aquatic bird populations (6). In contrast, outbreaks of HPAIV are extremely rare in wild birds (7). Although the role of wild birds in HPAIV maintenance remains controversial (8), the magnitude of the subtype H5N1 epidemics increased the demand for early recognition of potential threats to humans and poultry and an understanding of the natural history of AIV in wild birds. Consequently, surveillance of aquatic bird populations surged (9).

Although surveillance for AIV often uses state-of-the-art storage, transport and diagnostics, these must be underpinned by appropriate survey design, sampling, and interpretation in the context of the host population. In the wake of such rapid growth in surveillance, we reviewed the literature to determine a scientifically and statistically sound approach to the design, conduct, and interpretation of surveillance for AIV and other wildlife diseases.

## Current Surveillance

**Figure 1:**
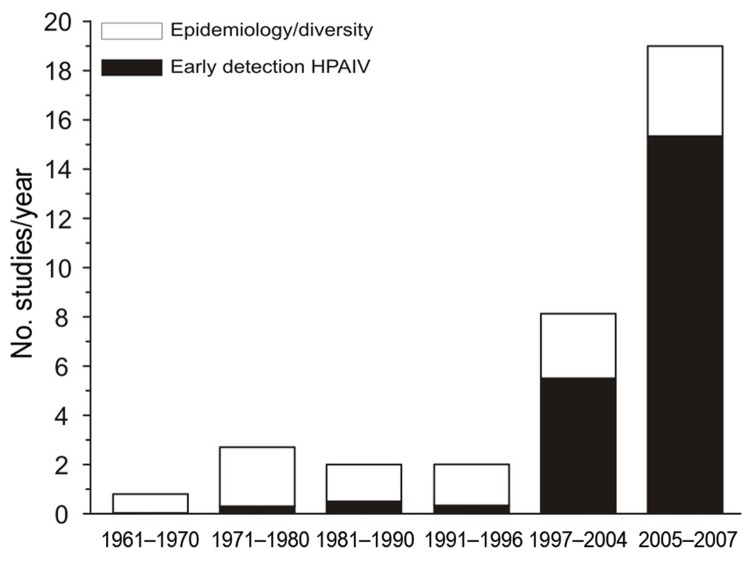
Average number of surveys of avian influenza in wild birds initiated per year in different awareness periods: each decade from the first discovery in 1961 until the outbreak of highly pathogenic avian influenza virus (HPAIV) (H5N1) in Asia in 1997; the period after the first outbreak, 1997–2004; and the period after mass deaths of wild birds from HPAIV (H5N1) (2005–2007). Black bar sections indicate studies citing the detection of contemporary HPAIV strains as one of the main aims of their survey are indicated in black; white bar sections indicate studies investigating other aspects of the wild bird–avian influenza system without mention of monitoring HPAIV.

We reviewed 191 published reports of surveillance in wild birds (Technical Appendix). The number of studies initiated per year rapidly increased after the first reports of HPAIV (H5N1) in Asia (Figure 1). All studies addressed 4 major lines of investigation: 1) early detection of HPAIVs; 2) ecology and epidemiology of LPAIV in host populations; 3) diversity and evolution of viral strains within wild birds; and 4) identification of the pathogens that infect individual birds or populations, often as part of multipathogen surveillance. Multiple aims can, and often are, addressed within the same surveillance program, albeit in a post hoc manner. However, identifying the aims in advance is vital, because what, when, and where to sample will critically depend on the purpose of the survey (10,11).

### Early Detection of HPAIV

More than half of the studies reviewed, and all but a handful initiated since the mass bird deaths in 2005–2006, cited early detection of HPAIV as one of the main goals of conducting the research (Figure 1). Such early warning systems question whether HPAIV exists in a population at a given location and point in time. The global rarity of HPAIV in wild birds and apparent clustering of such cases (7) present additional challenges to addressing this aim.

### Ecology and Epidemiology

Greater understanding of transmission cycles, reservoirs, and the role of wildlife in the dynamics of AIV invoke questions related to the epidemiology and ecology of the virus, including host range and spatial and temporal variation in infection (12,13). Elucidating such questions requires investigating not just presence or absence of infection in a specific host, but also prevalence over space and time.

### Viral Diversity

Influenza viruses are highly diverse and capable of rapid genetic alteration. Understanding the pathogenic and antigenic properties of AIVs circulating in the host population and the rate and direction of genetic alterations could become a powerful tool for identifying transmission parameters, reservoir populations (14), viral maintenance in the face of host immunity (12,15), and factors promoting disease emergence (10). Such information also facilitates compilation of comprehensive diagnostic reference panels and generation of potential vaccines (13). Investigation of variation in the viral population requires isolates that represent the entire circulating virus pool.

### Host Health

Almost 15% of the studies reviewed aimed to ascertain whether certain individuals or populations had been infected with AIV as part of broader health surveys within the context of conservation programs, or in an attempt to understand causes of death. Although these studies often have a predefined host population of interest, they are likely to be sensitive to the underlying spatial and temporal patterns of disease.

## Critical Assessment

To characterize the specific features required for rigorous wildlife disease surveillance, it is critical to highlight methods that encumber our current approach. Our assessment therefore aims to foster the development of more objective and scientifically sound disease surveillance networks.

### Maximizing Viral Yield

A successful surveillance program is often perceived as one that identifies a high number of positive samples. Moreover, exploitation of spatial, temporal, phylogenetic, and demographic differences in viral prevalence have been advocated to maximize the proportion of positive samples collected (12,16). Minimizing the number of negative samples is expedient from a laboratory perspective, particularly when labor-intensive virus isolation techniques are being used. However, a key tenet of surveillance is that the sampling scheme is representative: infection characteristics of the host population and genetic diversity of the viral population are sufficiently captured, and results can be interpreted on the basis of statistical probability (11,17). A study designed to maximize the number of positive samples by sampling historically high cohorts, populations, times, and locations can confirm the presence of the disease in the sampled cohort. However, such samples cannot be used to conclude the absence of AIV in the population or to estimate prevalence or diversity of circulating viral strains (17).

### Host Range

Although AIVs have been isolated from >100 species, several species from the orders Anseriformes (ducks, geese, and swans) and Charadriiformes (shorebirds) are thought to act as the reservoir community for AIV (6), primarily because AIVs have been most frequently isolated from these groups (9). Yet, surveillance is rarely representative of the diversity of wild birds or their relative abundance at the time and location of sampling. Considerable bias exists toward species that are easily caught or are present in accessible areas at high concentrations (9,13). Surveys that have included a wide range of species often obtained samples in a highly opportunistic manner, resulting in few species being sampled in reasonable numbers (12–13). For instance, despite sampling >56,000 birds in the Netherlands from 1998 to 2009, only 20 of the 174 species were sampled >300 times. Moreover, prevalence in a given species may vary over space and time. Although passerines have often been found negative for AIV, recent evidence suggests that, when sampled in or near waterfowl-rich bodies of water, a high proportion of individuals from 8 different passerine families show infection (18,19). Current surveillance may, therefore, overlook many potential reservoir or transient host species and their role in the introduction, transmission, maintenance and diversity of AIV.

### Temporal and Spatial Patterns

The prevalence of AIV infection has long been recognized to vary over time and space. Viruses have been most frequently isolated from duck populations in North America and Europe in late summer and early autumn (5,15,20), a pattern attributed to high concentrations of susceptible juvenile birds on premigratory staging grounds (4,6). Less frequent isolations from wintering populations have prompted suggestions that prevalence rapidly decreases over the course of autumn migration (21,22); thus, premigratory staging grounds in late summer and early autumn are considered the optimal time and location for conducting surveillance among waterfowl (16,23). Yet when samples have been collected elsewhere, high numbers of AIVs have been isolated in winter (21,24), spring (20), and summer (25). Several positive samples from birds in the tropics (26) have also been found, including unexpectedly high numbers in tropical Africa (27). The temporal and spatial bias in existing surveillance may therefore result in delayed detection of novel strains or an incomplete understanding of AIV transmission, maintenance, diversity, and evolution.

### Age-dependent Patterns

Pioneering work by Hinshaw et al. (4) found significantly higher prevalence of AIV infection among juvenile birds than among contemporaneously sampled adult birds, leading to the suggestion that immunological naivety may make juvenile birds a high-risk group within waterfowl populations. Emphasis has subsequently been placed on sampling juvenile birds; accounting for ≈80% in some recent surveys. However, wild bird populations are rarely composed of >80% juvenile birds, and numerous infected adults have also been found (4,24). Given that recent experimental results indicate that age at the time of infection might also affect the extent of viral shedding (28), different age cohorts may play different roles in the introduction, transmission, maintenance, and diversity of AIVs.

### Site of Infection

**Figure 2:**
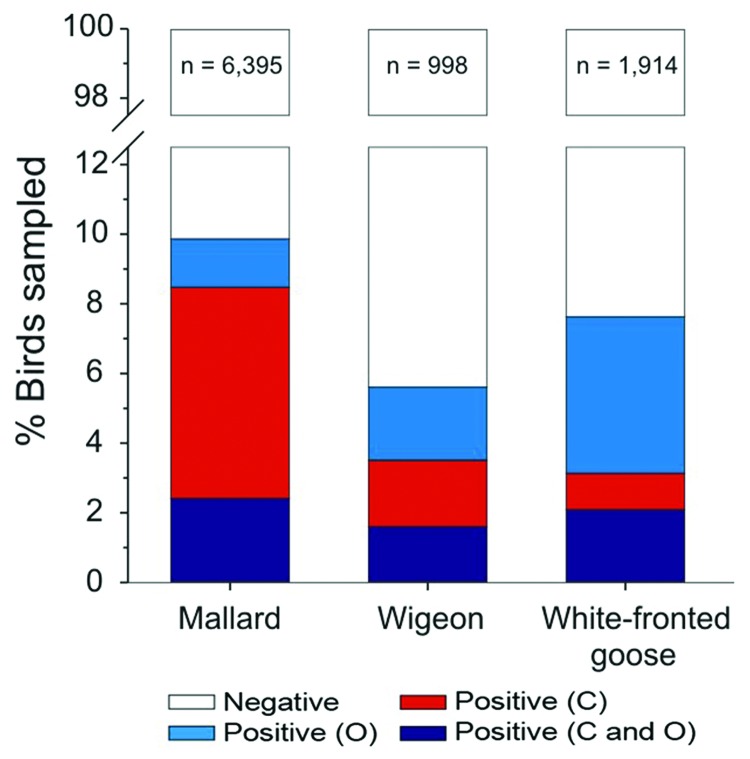
Proportion of wild mallards (*Anas platyrhynchos*), Eurasian wigeons (*Anas penelope*), and white-fronted geese (*Anser albifrons*) positive for low-pathogenicity avian influenza virus when sampled in the cloaca (C) and the oropharynx (O), the Netherlands, September 2006–March 2009.

AIVs replicate in the gastrointestinal tract (sampled by swabbing the cloaca or collecting droppings) and in the respiratory tract (sampled by swabbing the oropharynx) (16). Individual mallards (*Anas platyrhynchos*) have historically shown higher detection probability from cloacal c.f. oropharyngeal swabs (*29*; Figure 2). Accordingly, 61% of studies investigating contemporary infection sampled the gastrointestinal tract alone. However, the site of infection may differ between species. As part of ongoing surveillance (21,29), free-living Eurasian wigeons (*Anas penelope*) showed no difference in detection probability between the cloacal and oropharyngeal swabs (p>0.05, McNemar test; Figure 2). In contrast, white-fronted geese (*Anser albifrons*) were roughly 2× as likely to have infection detected in the oropharynx (6.58%; 95% confidence interval 6.57–6.59) than in the cloaca (3.13%; 95% confidence interval 3.13–3.14; p <0.001); ≈60% of the infected birds were positive by oropharynx sample alone (Figure 2). Together with the apparent oropharynx affinity of HPAIV (H5N1) in experimental and natural infections (30), these findings have ramifications for the quantification of viral diversity, prevalence of infection, or absence of AIV.

### Disease-free Populations and Prevalence Estimates

In general, survey sample sizes must be sufficiently large to draw appropriate inferences, and interpretations of AIV in wild birds based on many current sampling schemes may be hampered due to the limited number of samples collected (9). Studies have often concluded that AIV, particularly HPAIV (H5N1), was absent from a certain population or location. Infected birds may indeed have been present, but at a prevalence below the level of detection of the study (17). Only 3 of the studies that reported negative findings acknowledged a detection limit, yet such information is crucial to screening for HPAIV incursion. Similarly, 81 (42%) of the articles reviewed explicitly reported prevalence or seroprevalence; however, just 3 of these accounted for the uncertainty of their estimates (i.e., confidence limits). Such reports have fostered an impression that prevalence is a fixed property of a given host population, rather than a dynamic quantity, potentially influenced by many temporal, geographic, and biological interactions.

### Utility of Birds Found Dead

Many surveillance programs aimed at the early detection of HPAIV (H5N1) focus on collections from sick or dead birds, often without surveillance of the living avian population (31). Although finding an HPAIV (H5N1) infection is statistically more likely in birds found dead (31), the absence of dead birds (or infection in dead birds) does not indicate freedom from disease. Dead birds fail to provide information on any animals that survived the infection, any animals that were not infected, or any viruses that were not lethal (30). Moreover, large numbers of carcasses may go undetected or unreported (10).

### Screening for Only the Current Strain of Interest

Recently, some studies have only screened for H5 strains. Yet, none of the known genotypes can be ruled out as potential candidates for future pandemics. Additional information on all circulating gene segments is preferable as a novel-incursion warning system and in the broader context of AIV ecology, epidemiology, and evolution, particularly because no additional sample collection is necessary.

## A Way Forward?

Although ≈50 years have passed since AIVs were first detected in wild birds, research is still in the exploratory phase, primarily because sampling wild animals is logistically challenging and expensive and techniques for high-throughput molecular surveillance have only recently become available. Wildlife disease surveillance regularly involves limited samples obtained in various ways that are already readily available, such as ornithologist-captured and hunter-collected birds. Although these methods of convenience sampling are often assumed to be representative of a population, sampling biases (most notably selection bias) do occur, making it difficult to develop statistically valid estimates of disease absence or prevalence, regardless of how many birds are sampled.

Our critique illustrates that to build on the findings of existing surveillance a scientifically sound approach is required. A study’s aims need to be clearly identified at the outset, and appropriately designed sampling regimes and diagnostic techniques must be used. The global distribution of AIV and its avian hosts presents a major hurdle for such hypothesis-based research, making it difficult for individual research groups to tackle these questions in isolation. Our review highlights the need for global collation of existing wild bird AIV data and infrastructure, as well as the pooling of expertise and resources between epidemiologists, ornithologists, geneticists, and conservation organizations to unravel the complex interactions among diverse host and viral populations and the environments they utilize. Many such international initiatives exist in principle; however, there are currently several challenges in terms of data coverage, compatibility, management, and ownership. The following section outlines key considerations pertaining to the design, implementation, and interpretation of local surveys that could ameliorate data coverage and compatibility problems, paving the way for increasingly integrated studies of AIV and other wildlife diseases.

## Sampling Unit

### Target Virus

Particular strains, especially those with a history of HPAIV potential (H5 and H7), are of greatest interest when screening for HPAIV (16). However, screening for other virus subtypes by virus isolation, or targeting the matrix gene segment in molecular-based diagnostics, will simultaneously enhance our ecologic, epidemiologic, and virologic understanding of AIV.

### Dead or Alive

Birds found dead may indicate rapid changes in host range, geographic range, viral pathogenicity, or disease emergence, and as such warrant swift investigation. However, to clarify the presence or absence of HPAIV, as well as trends in LPAIV presence, prevalence, and circulating strains, such surveys should be paired with active surveillance of the living wild bird population.

### Sampling Site within the Bird

Viral strains of different host origin may differ in their affinity for either the digestive or respiratory tract and may also differ between different host species. Sampling the cloaca/feces and oropharynx is therefore desirable when screening wild birds. Such differences also exemplify the need for experimental clarification of tract affinity and how this may influence interpretations based on a single sample type (e.g., droppings).

## Which Populations Should Be Sampled?

### Target Population

With >10,000 species of birds worldwide, careful selection of a local target population is critical to the design of any surveillance program. Because the prevalence of infection is generally low (requiring large sample sizes) and can vary over time and between locations within a species, it is difficult to make an initial assessment of the most important species to target on the basis of virus detection alone. Each of the surveillance aims outlined above may be most appropriately addressed by considering 1) populations with evidence of previous infection, or ecologic potential for infection (32), on the basis of not only existing literature and conventional monitoring but also serosurveillance in a large number of locally and regionally abundant species; and 2) Evidence of contemporary AIV infection in populations that were identified in step 1, and species in which AIV has historically been detected (for comparative purposes). Surveillance for emergent HPAIV may also benefit from targeting species displaying natural histories of interest, including species that link wild and human/agricultural populations or disparate locations.

Serologic studies have great potential for enhancing wildlife disease surveillance and understanding. However, in isolation, cross-sectional observations of seroprevalence provide insufficient information to interpret the degree to which a population has been infected with AIV. Without age specificity, high seroprevalence may indicate a recent outbreak of infection or long-term antibody maintenance rather than persistence of AIV infection in the population (14,16). Moreover, low seroprevalence may result from a high mortality rate among infected birds, a long time interval between infection and sampling, or species-specific differences in the sensitivity or specificity of the antibody diagnostics. Explicit interpretation of seroprevalence calls for age-specific sampling, longitudinal observations, understanding of the underlying epidemiologic dynamics, and experimental validation of antibody diagnostics.

### Individual Birds within Populations

Within each species, infection may depend on multiple factors, including age and prior exposure to AIV (4), gender (33), and even nutrition or social status (8). Given that most capture methods inherently result in biases within these cohorts, a population should ideally be sampled to account for these differences. Experimental validation of such interindividual differences in infection could greatly enhance the design and interpretation of surveillance.

## When, Where, and How Often to Sample?

When and where sampling is conducted will critically depend on the question at hand and should be representative of the biology of the hosts of interest. Single time or location studies may be sufficient to inform of novel incursions of HPAIV (Table) and may therefore be best matched to times/locations with a high risk for wild bird–poultry interaction. Changes in climatic conditions, host population dynamics, and host population immunity are likely relevant to understanding the ecology, epidemiology, and evolution of AIV in its natural host(s) (34). Enhancing our knowledge in these areas will require information from before, during, and after infection from ecologically connected populations (35), often over longer periods and across large spatial scales when studying migratory birds (36). Coordinated local surveys, both along flyways and over time, will greatly enhance these efforts.

**Table:** Data requirements for assessment of major questions regarding avian influenza in wild birds*

Aim	Type of question	Geographic range	Temporal range	Frequency
Early detection of HPAIV	Presence/absence	Local/regional	Period when birds present	Approximately weekly (average infection duration)
Ecology and epidemiology	Comparative prevalence	Local to flyway, depending on the process in question	1 to many epidemic seasons (multiple times/year)	Weekly to monthly (multiple times before, during, and after an epidemic)
Diversity and evolution	Comparative prevalence (of viral strains)	Flyway to global	Decades (multiple times/year repeated for multiple years)	Monthly to seasonally

*Larger-scale studies can be compiled over large geographic areas from relevant local surveys that are methodologically comparable and over long periods from relevant annual surveys that are likewise methodologically comparable. HPAIV, highly pathogenic avian influenza virus.

## How Many Individual Birds Should Be Sampled?

**Figure 3:**
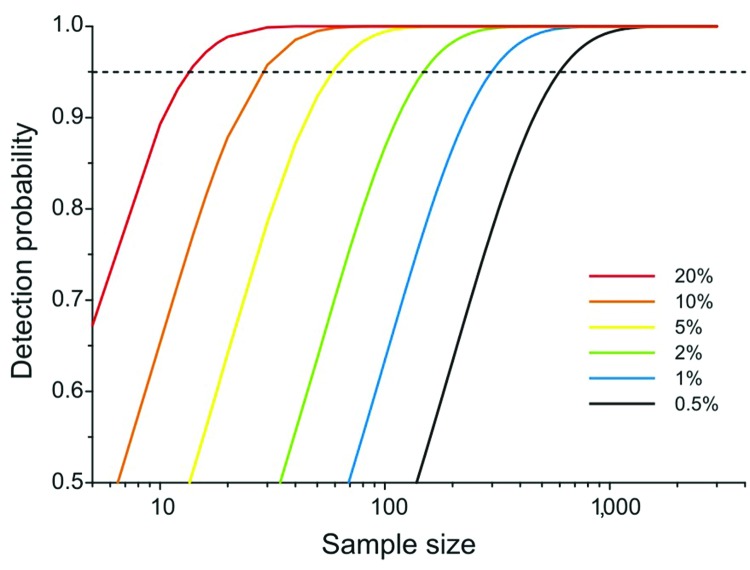
Probability of detecting >1 individual bird infected with avian influenza virus from a given number of samples selected at random from an extremely large population in which individual birds are infected at random at different prevalence levels. Although this nominal minimum detectable prevalence assumes binomial sampling, it can also be used for gaining a rough quantitative estimate of the minimum number of samples required before embarking on a surveillance program.

**Figure 4:**
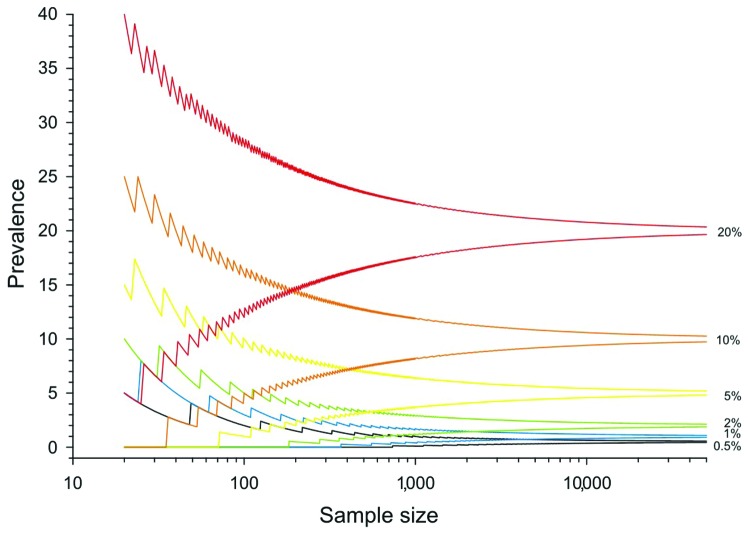
The 95% confidence intervals for prevalence in an independent population for a given number of samples, derived from the binomial distribution. Confidence intervals depend on the number of samples taken and unbiased prevalence of infection; they should be calculated and reported along with prevalence estimates when reporting surveillance results.

As prevalence decreases, an increasingly large number of birds need to be sampled to detect contemporary infection (Figure 3, Figure 4). Deciding just how many is critically dependent on the study aim, with a clear distinction between surveys that aim to substantiate freedom from infection (presence or absence), and those that are designed to provide an estimate of disease prevalence.

### Presence/Absence

In practice, it is not possible to confirm disease freedom in a large population by any direct observational method. Instead, appropriate sampling and analysis can demonstrate that at that time and location, prevalence was below a nominal detection threshold (Technical Appendix) (17). Although this nominal minimum detectable prevalence assumes binomial sampling, it can also be used for gaining a rough quantitative estimate of the minimum number of samples required before embarking on a surveillance program (Figure 3; Technical Appendix). Given that information on the absence of pathogens is crucial to understanding disease dynamics (10), postsurveillance reporting of such maximum undetected prevalence is highly desirable for all studies with negative findings.

### Prevalence

The proportion of positive findings among a given number of samples is rarely sufficiently precise to inform population prevalence. Thus, the confidence intervals of any observed proportion should be calculated and reported alongside any prevalence estimates when reporting surveillance results. Such confidence limits depend on the number of samples taken and the underlying true (unbiased) prevalence of infection (Figure 4).

## Achieving Effective Surveillance

Each of the points above highlight the need for surveillance that captures the underlying temporal, spatial, demographic, and phylogenetic variation in the wild bird population, often requiring detailed information on host population size, density, demographic structure, rates of recruitment and attrition, habitat utilization, and species composition. However, wildlife surveillance is also faced with substantial logistical and financial constraints. Effective surveillance, therefore, requires a compromise between sampling that is based on probability and the constraints of sample collection, transport and analysis, the details of which will depend on the specific objectives of the survey. To this end, it is critical to have active, investigator-defined surveillance designs based on probability on a larger scale while using convenience sampling within these units (11). For instance, probability methods could be used to plan the species, locations, and months of the year to sample, and a certain number of individual birds within these units could be sampled by ornithologists and hunters, with additional top-up sampling where necessary. Such convenience-within-probability surveillance could provide statistically valid estimates of disease absence and prevalence by reducing the effect of bias generated by sampling on a first-come-first-served basis. It facilitates stipulation of an upper limit to the use of convenience samples, allowing targeted allocation of limited sampling, diagnostic, and financial resources.

To employ such convenience-within-probability surveillance, samples will often need to be collected from times, places, and species that are not currently covered by ornithologists and hunters. Preferably, individual birds should be sampled to confirm species, gender, age, and body mass, and sampling of digestive and respiratory tracts. However, when it is logistically and/or financially difficult to capture live birds several alternatives exist. Swabbing of fresh, species-specific feces is 1 method for collecting a regulated number of samples (16). Species should be identified through careful presampling observation of flocks, or, when sampling mixed-species flocks, through DNA barcoding of the fecal samples (37). Given that AIV can be detected from the same nucleic acid extract used in species identification (37), and substantially more samples can be collected at a much higher frequency than traditional trapping methods, dropping samples may greatly enhance our capacity to detect AIV in the population. Other, more proximate surveillance methods include sampling surface water that is, has been, or is about to be inhabited by wild birds (16), as well as regular sampling of sentinel species (38). Both methods are likely to yield insight into infection in the broader host population (16), although their usefulness for understanding infection in specific populations must be carefully assessed.

## Conclusions

Surveillance for wildlife diseases is an inherently arduous task. However, as the vanguard of our understanding of these diseases, surveillance warrants a scientific approach. To make major inroads into the broader understanding of AIV ecology, epidemiology, and evolution, as well as risks associated with HPAIV, an integrated sampling strategy with clearly defined aims and appropriate methods is required. The financial and logistical constraints of covering vast spatial and temporal scales call for concerted efforts among our combined virologic, ecologic, and genetic expertise.

## Supplementary Material

Technical Appendix Source References and Formulas for Estimating Minimum Detectable Prevalence.
