# Supplementary material for: Surveillance of Wild Birds for Avian Influenza Virus
Source: Emerg Infect Dis. 2010 Dec;16(12):1827–34. doi: 10.3201/eid1612.100589 (PMC3294547; doi:10.3201/eid1612.100589)
Supplement: Technical Appendix — Source References and Formulas for Estimating Minimum Detectable Prevalence. [file 10-0589-Techapp_19p.pdf]

Article DOI: 10.3201/eid1612.100589

Surveillance of Wild Birds for Avian Influenza Virus

Technical Appendix

Source References

The articles reporting avian influenza surveillance in wild birds included in this review were obtained by searching for [influenza OR ortho*] AND [virus*] AND [surve* OR monitor* OR sampl*] AND [wild* OR free-living OR “free living” OR feral OR migratory OR resident] AND [avian OR bird* OR waterfowl] on both Pubmed and Web of Knowledge on March 18, 2010. All studies were initiated between 1961 and 2007. We refined our list by including only peer-reviewed articles, and by excluding studies on captive individuals, domesticated species, or duplicate reports from the same study, resulting in the following 191 articles:

Abolnik C, Cornelius E, Bisschop SPR, Romito M, Verwoerd D. Phylogenetic analyses of genes from South African LPAI viruses isolated in 2004 from wild aquatic birds suggests introduction by Eurasian migrants. In: Schudel A, Lombard M, editors. OIE/FAO International Scientific Conference on Avian Influenza. Basel: Karger; 2006. p. 189–99.

Al-Attar MY, Danial FA, Al-Baroodi SY. Detection of Antibodies Against Avian Influenza Virus in Wild Pigeons and Starlings. Journal of Animal and Veterinary Advances. 2008;7(4):448–9.

Alekseev AY, Sharshov KA, Zolotykh SI, Drozdov IG, Shestopalov AM. Monitoring of influenza A viruses in synanthropic birds in South of Western Siberia in H5N1 epizootic and postepizootic period. Infection Genetics and Evolution. 2008 Jul;8(4):S32.

Alfonso CP, Cowen BS, Vancampen H. Influenza-a Viruses Isolated from Waterfowl in 2 Wildlife Management Areas of Pennsylvania. Journal of Wildlife Diseases. 1995 Apr;31(2):179–85.

Anraku MM, de Godoy F, Oscar MG, de Fario WC. Avian influenza in wild Brazilian birds. I. Serological survey by means of immunodiffusion [in Portuguese]. Revista do Instituto de Medicina Tropical de São Paulo. 1977;19(4):237–40.

Arenas A, Carranza J, Perea A, Miranda A, Maldonado A, Hermoso M. Type-A influenza-viruses in birds in southern spain - serological survey by enzyme-linked-immunosorbent-assay and hemagglutination inhibitin tests. Avian Pathology. 1990;19(3):539–46.

Artois M, Manvell R, Fromont E, Schweyer JB. Serosurvey for Newcastle disease and avian influenza A virus antibodies in great cormorants from France. Journal of Wildlife Diseases. 2002;38(1):169–71.

Astorga RJ, Leon L, Cubero MJ, Arenas A, Maldonado A, Tarradas MC, et al. Avian Influenza in Wild Waterfowl and Shorebirds in the Donana-National-Park - Serological Survey Using the Enzyme-Linked-Immunosorbent-Assay. Avian Pathology. 1994;23(2):339–44.

Austin FJ, Hinshaw VS. The isolation of Influenza-A viruses and paramyxoviruses from feral ducks in New Zealand. Australian Journal of Experimental Biology and Medical Science. 1984;62:355–60.

Bahl AK, Pomeroy BS, Easterday BC, Mangundimedjo S. Isolation of type A influenza viruses from migrating waterfowl along the Mississippi flyway. Journal of Wildlife Diseases. 1975;11:360–3.

Barbic L, Turk N, Zupancic Z, Kovac S, Stojevic Z, Drazenovic V, et al. Antibodies against human influenza viruses in sentinel duck flocks in the ornithological reserve Kopacki rit in Croatia. Veterinarski Arhiv. 2009 Nov-Dec;79(6):573–82.

Barral M, Alvarez V, Juste RA, Agirre I, Inchausti I. First case of highly pathogenic H5N1 avian influenza virus in Spain. BMC Vet Res. 2008 Dec;4:5.

Baumeister E, Leotta G, Pontoriero A, Campos A, Monalti D. Serological evidence of influenza A virus infection in Antarctica migratory birds. International Congress Series. 2004;1263:737–40.

Becker WB. Isolation and classification of tern virus - Influenza virus A/Tern/South Africa/1961. Journal of Hygiene. 1966;64(3):309–20.

Betakova T, Marcin J, Kollerova E, Molcany T, Dravecky M, Nemeth J, et al. Detection of an influenza virus in wild waterbirds migrating through Slovakia in autumn 2004. Acta Virologica. 2005;49(4):287–9.

Bolte AL, Lutz W, Kaleta EF. [Investigations on the occurrence of ortho- and paramyxovirus infections among free living greylag geese (*Anser anser* Linne, 1758)] [in German]. Zeitschrift Fur Jagdwissenschaft. 1997;43(1):48–55.

Bonner BM, Lutz W, Jager S, Redmann T, Reinhardt B, Reichel U, et al. Do Canada geese (*Branta canadensis* Linnaeus, 1758) carry infectious agents for birds and man? European Journal of Wildlife Research. 2004 Jul;50(2):78–84.

Busquets N, Alba A, Napp S, Sánchez A, Serrano E, Rivas R, et al. Influenza A virus subtypes in wild birds in North-Eastern Spain (Catalonia). Virus Research. 2010;[Epub ahead of print].

Campitelli L, Mogavero E, De Marco MA, Delogu M, Puzelli S, Freeza F. Influenza surveillance in birds in Italy (1999-2002): first evidence in the wild avian reservoir of direct virus precursor of strains circulating in commercial poultry. International Congress Series. 2004;1263:766–70.

Cattoli G, Terregino C, Guberti V, De Nardi R, Drago A, Salviato A, et al. Influenza virus surveillance in wild birds in Italy: Results of laboratory investigations in 2003-2005. Avian Diseases. 2007;51(1):414–6.

Chang CM, Lebarbenchon C, Gauthier-Clerc M, Le Bohec C, Beaune D, Le Maho Y, et al. Molecular surveillance for avian influenza A virus in king penguins (*Aptenodytes patagonicus*). Polar Biol. 2009 Apr;32(4):663–5.

Charlton KG. Antibodies to selected disease agents in translocated wild turkeys in California. Journal of Wildlife Diseases. 2000 Jan;36(1):161–4.

Chen HX, Shen HG, Li XL, Zhou JY, Hou YQ, Guo JQ, et al. Seroprevalance and identification of influenza A virus infection from migratory wild waterfowl in China (2004-2005). Journal of Veterinary Medicine Series B-Infectious Diseases and Veterinary Public Health. 2006 May;53(4):166–70.

Cheng MC, Wang CH, Kida H. Influenza A virological surveillance in feral waterfowl in Taiwan from 1998 to 2002. International Congress Series. 2004;1263:745–8.

Chernetsov I, Slepushkin AN, L'vov DK, Braude NA, Gavrilov AE. [Isolation of influenza virus from Chlidonias nigra and serologic examination of the birds for antibodies to influenza virus] [in Russian]. Voprosy Virusologii. 1980(1):35–40.

Dalessi S, Hoop R, Engels M. The 2005/2006 avian influenza monitoring of wild birds and commercial poultry in Switzerland. Avian Diseases. 2007;51(1):355–8.

D'Amico VL, Bertellotti M, Baker AJ, Diaz LA. Exposure of Red Knots (*Calidris canutus rufa*) to select avian pathogens; Patagonia Argentina. Journal of Wildlife Diseases. 2007;43(4):794–7.

Dasen CA, Laver WG. Antibodies to influenza viruses (including human A2/Asian/57 strain) in sera from Australian shearwaters (*Puffinus pacificus*). Bulletin of the World Health Organization. 1970;42(6):885–9.

Davidson WR, Yoder HW, Brugh M, Nettles VF. Serological monitoring of eastern wild turkeys for antibodies to Mycoplasma spp and avian influenza-viruses. Journal of Wildlife Diseases. 1988 Apr;24(2):348–51.

De Marco MA, Campitelli L, Delogu M, Raffini E, Foni E, di Trani L, et al. Serological evidences showing the involvement of free-living pheasants in the influenza ecology. Italian Journal of Animal Science. 2005;4(3):287–91.

De Marco MA, Foni E, Campitelli L, Delogu M, Raffini E, Chiapponi C, et al. Influenza virus circulation in wild aquatic birds in Italy during H5N2 and H7N1 poultry epidemic periods (1998 to 2000). Avian Pathology. 2005 Dec;34(6):480–5.

De Marco MA, Foni E, Campitelli L, Raffini E, Delogu M, Donatelli I. Long-term monitoring for avian influenza viruses in wild bird species in Italy. Veterinary Research Communications. 2003 Sep;27:107–14.

De Marco MA, Foni GE, Campitelli L, Raffini E, Trani LD, Delogu M, et al. Circulation of Influenza Viruses in Wild Waterfowl Wintering in Italy During the 1993–99 Period: Evidence of Virus Shedding and Seroconversion in Wild Ducks. Avian Diseases. 2003;47:861–6.

De Nardi R, Terregino C, Cattoli G, Toffan A, Beato MS, Guberti V, et al. Preliminary results of an influenza surveillance in wild birds, game birds, domestic ducks and geese in North Eastern Italy. Italian Journal of Animal Science. 2005 Jul-Sep;4(3):292–5.

Deem SL, Noss AJ, Cuellar RL, Karesh WB. Health evaluation of free-ranging and captive blue-fronted Amazon parrots (*Amazona aestiva*) in the Gran Chaco, Bolivia. Journal of Zoo and Wildlife Medicine. 2005 Dec;36(4):598–605.

Deibel R, Emord DE, Dukelow W, Hinshaw VS, Wood JM. Influenza Viruses and Paramyxoviruses in Ducks in the Atlantic Flyway, 1977-1983, including an H5N2 Isolate Related to the Virulent Chicken Virus. Avian Diseases. 1985;29(4):970–85.

Dormitorio TV, Giambrone JJ, Guo K, Hepp GR. Detection and characterization of avian influenza and other avian paramyxoviruses from wild waterfowl in parts of the southeastern United States. Poultry Science. 2009;88(4):851–5.

Douglas KO, Lavoie MC, Kim LM, Afonso CL, Suarez DL. Isolation and Genetic Characterization of Avian Influenza Viruses and a Newcastle Disease Virus from Wild Birds in Barbados: 2003-2004. Avian Diseases. 2007;51(3):781–7.

Downie JC, Hinshaw V, Laver WG. The ecology of influenza: Isolation of type ‘A’ influenza viruses from Australian pelagic birds. Australian Journal of Experimental Biology and Medical Science. 1977;55(DEC):635–43.

Downie JC, Laver WG. Isolation of a Type a Influenza-Virus from an Australian Pelagic Bird. Virology. 1973;51(2):259–69.

Ducatez MF, Tarnagda Z, Tahita MC, Sow A, de Landtsheer S, Londt BZ, et al. Genetic characterisation of HPAI (H5N1) viruses from poultry and wild vultures, Burkina Faso. Emerging Infectious Diseases. 2007;13(4):611–3.

Dusek RJ, Bortner JB, DeLiberto TJ, Hoskins J, Franson JC, Bales BD, et al. Surveillance for High Pathogenicity Avian Influenza Virus in Wild Birds in the Pacific Flyway of the United States, 2006–2007. Avian Diseases. 2009;53:222–30.

Easterday BC, Trainer DO, Tumova B, Pereira HG. Evidence of Infection with Influenza Viruses in Migratory Waterfowl. Nature. 1968;219(5153):523–4.

Ellström P, Latorre-Margalef N, Griekspoor P, Waldenstrom J, Olofsson J, Wahlgren J, et al. Sampling for low-pathogenic avian influenza A virus in wild Mallard ducks: Oropharyngeal versus cloacal swabbing. Vaccine. 2008 Aug;26(35):4414–6.

Fereidouni SR, H. BM, Starick E, Werner O, Amini H, Modirrousta H, et al. Serological Monitoring of Avian Influenza in Migratory Birds of Iran. Archives of Razi Institute. 2005;60:11–9.

Fereidouni SR, Werner O, Starick E, Beer M, C. HT, Aghakhan M, et al. Avian influenza virus monitoring in wintering waterbirds in Iran, 2003-2007. Virology Journal. 2010;[Epub ahead of print].

Ferrazzi V, Martin AM, Lelli D, Gallazzi D, Grilli G. Microbiological and serological monitoring in hooded crow (*Corvus corone cornix*) in the Region Lombardia, Italy. Italian Journal of Animal Science. 2007;6(3):309–12.

Ferro PJ, El-Attrache J, Fang XW, Rollo SN, Jester A, Merendino T, et al. Avian influenza surveillance in hunter-harvested waterfowl from the Gulf Coast of Texas (November 2005-January 2006). Journal of Wildlife Diseases. 2008 Apr;44(2):434–9.

Fujimoto Y, Ito H, Shivakoti S, Nakamori J, Tsunekuni R, Otsuki K, et al. Avian Influenza Virus and Paramyxovirus Isolation from Migratory Waterfowl and Shorebirds in San-In District of Western Japan from 2001 to 2008. Journal of Veterinary Medical Science. 2010;[Epub ahead of print].

Gaidet N, Dodman T, Caron A, Balança G, Desvaux S, Goutard F, et al. Avian influenza viruses in water birds, Africa. Emerging Infectious Diseases. 2007;13(4):626–9.

Gaidet N, Dodman T, Caron A, Balança G, Desvaux S, Goutard F, et al. Influenza Surveillance in Wild Birds in Eastern Europe, the Middle East, and Africa: Preliminary Results from an Ongoing FAO-led Survey. Journal of Wildlife Diseases. 2007;43(3):S22–S28.

Ghersi BM, Blazes D, Icochea E, Gonzalez RI, Kochel T, Tinoco Y, et al. Avian Influenza in wild birds from the central coast of Peru. Emerging Infectious Diseases. 2009;15(6):935.

Gilbert M. Disease Surveillance: Avian Influenza in Migratory Birds in Mongolia - Results of Extensive Surveys – 2006. Ulaanbaatar, Mongolia.: Wildlife Conservation Society; 2007.

Globig A, Baumer A, Revilla-Fernandez S, Beer M, Wodak E, Fink M, et al. Ducks as Sentinels for Avian Influenza in Wild Birds. Emerging Infectious Diseases. 2009;15(10):1633–6.

Globig A, Starick E, Werner O. Isolation of avian influenza from migratory waterfowl in Germany. Results of a two year study [in German]. Berliner Und Munchener Tierarztliche Wochenschrift. 2006 Mar-Apr;119(3-4):132–9.

Goujgoulova G, Oreshkova N. Surveillance on avian influenza in Bulgaria. Avian Diseases. 2007;51:382–6.

Graham DA, German A, Abernethy D, McCullough SJ, Manvell RJ, Alexander DJ. Isolation of ortho- and paramyxoviruses from wild birds in Northern Ireland during the 1997 Newcastle disease epizootic. Veterinary Record. 1999;145(1):20–1.

Graves IL. Influenza-viruses in birds of the Atlantic Flyway. Avian Diseases. 1992 Jan-Mar;36(1):1–10.

Gronesova P, Ficova M, Mizakova A, Kabat P, Trnka A, Betakova T. Prevalence of avian influenza viruses, *Borrelia garinii*, *Mycobacterium avium*, and *Mycobacterium avium* subsp paratuberculosis in waterfowl and terrestrial birds in Slovakia, 2006. Avian Pathology. 2008;37(5):537–43.

Gronesova P, Kabat P, Trnka A, Betakova T. Using nested RT-PCR analyses to determine the prevalence of avian influenza viruses in passerines in western Slovakia, during summer 2007. Scandinavian Journal of Infectious Diseases. 2008;40(11-12):954–7.

Halvorson DA, Kelleher CJ, Senne DA. Epizootiology of Avian Influenza - Effect of Season on Incidence in Sentinel Ducks and Domestic Turkeys in Minnesota. Applied and Environmental Microbiology. 1985;49(4):914–9.

Hanson BA, Luttrell MP, Goekjian VH, Niles L, Swayne DE, Senne DA, et al. Is the occurrence of avian influenza virus in Charadriformes species and location dependent? Journal of Wildlife Diseases. 2008;44(2):351–61.

Hanson BA, Stallknecht DE, Swayne DE, Lewis LA, Senne DA. Avian Influenza Viruses in Minnesota Ducks During 1998–2000. Avian Diseases. 2003;47:867–71.

Hanson BA, Swayne DE, Senne DA, Lobpries DS, Hurst J, Stallknecht DE. Avian influenza viruses and paramyxoviruses in wintering and resident ducks in Texas. Journal of Wildlife Diseases. 2005 Jul;41(3):624–8.

Happold JR, Brunhart I, Schwermer H, Stärk KDC. Surveillance of H5 Avian Influenza Virus in Wild Birds Found Dead. Avian Diseases. 2008;52:100–5.

Hars J, Ruette S, Benmergui M, Fouque C, Fournier JY, Legouge A, et al. The epidemiology of the highly pathogenic H5N1 avian influenza in mute swan (*Cygnus olor*) and other Anatidae in the Dombres Region (France), 2006. Journal of Wildlife Diseases. 2008;44(4):811–23.

Haynes L, Arzey E, Bell C, Bucanan N, Burgess G, Cronan V, et al. Australian surveillance for avian influenza viruses in wild birds between July 2005 and June 2007. Australian Veterinary Journal. 2009;87:266–72.

Heard DJ, Mulcahy DM, Iverson SA, Rizzolo DJ, Greiner EC, Hall J, et al. A blood survey of elements, viral antibodies, and hemoparasites in wintering Harlequin Ducks (*Histrionicus histrionicus*) and Barrow's Goldeneyes (*Bucephala islandica*). Journal of Wildlife Diseases. 2008 Apr;44(2):486–93.

Hesterberg U, Harris K, Stroud D, Guberti V, Busani L, Pittman M, et al. Avian Influenza surveillance in wild birds in the European Union in 2006. Influenza and Other Respiratory Viruses. 2009;3(1):1–14.

Hinshaw VS, Nettles VF, Schorr LF, Wood JM, Webster RG. Influenza virus surveillance in waterfowl in Pennsylvania after the H5N2 avian outbreak. Avian Diseases. 1986 Jan-Mar;30(1):207–12.

Hinshaw VS, Webster RG, Turner B. The perpetuation of orthomyxoviruses and paramyxoviruses in Canadian waterfowl. Canadian Journal of Microbiology. 1980;26:622–9.

Hinshaw VS, Wood JM, Webster RG, Deibel R, Turner B. Circulation of Influenza-Viruses and Paramyxoviruses in Waterfowl Originating from two Different Areas of North-America. Bulletin of the World Health Organization. 1985;63(4):711–9.

Hlinak A, Muhle RU, Werner O, Globig A, Starick E, Schirrmeier H, et al. A virological survey in migrating waders and other waterfowl in one of the most important resting sites of Germany. Journal of Veterinary Medicine Series B-Infectious Diseases and Veterinary Public Health. 2006 Apr;53(3):105–10.

Honda E, Kida H, Yanagawa R, Matsuura Y, Yagyu K, Tsuji M, et al. Survey of influenza viruses in feral birds in 1979 and isolation of a strain possessing Hav6Nav5 from cloaca of an eastern dunlin. Japanese Journal of Veterinary Research. 1981;29(3-4):83–7.

Hopkins BA, Skeeles JK, Houghten GE, Slagle D, Gardner K. A survey of infectious-diseases in wild turkeys (*Meleagridis gallopavo silvestris*) from Arkansas. Journal of Wildlife Diseases. 1990;26(4):468–72.

Hua Y-P, Chai H-L, Yang S-Y, Zeng X-W, Sun Y. Primary survey of avian influenza virus and Newcastle disease virus infection in wild birds in some areas of Heilongjiang Province, China. Journal of Veterinary Science. 2005;6(4):311–5.

Hurt AC, Hansbro PM, Selleck P, Olsen B, Minton C, Hampson AW, et al. Isolation of avian influenza viruses from two different transhemispheric migratory shorebird species in Australia. Archives of Virology. 2006 Nov;151(11):2301–9.

Iashkulov KB, Shchelkanov MI, L'Vov SS, Dzhambinov SD, Galkina IV, Fediakina IT, et al. [Isolation of influenza virus A (Orthomyxoviridae, Influenza A virus), Dhori virus (Orthomyxoviridae, Thogotovirus), and Newcastle's disease virus (Paromyxoviridae, Avulavirus) on the Malyi Zhemchuzhnyi Island in the north-western area of the Caspian Sea] [in Russian]. Voprosy Virusologii. 2008;53(3):34–8.

Ip HS, Flint PL, Franson JC, Dusek RJ, Derksen DV, Gill RE, et al. Prevalence of Influenza A viruses in wild migratory birds in Alaska: Patterns of variation in detection at a crossroads of intercontinental flyways. Virology Journal. 2008 Jun;5:71.

Ito T, Okazaki K, Kawaoka Y, Takada A, Webster RG, Kida H. Perpetuation of Influenza-a Viruses in Alaskan Waterfowl Reservoirs. Archives of Virology. 1995;140(7):1163–72.

Iverson SA, Takekawa JY, Schwarzbach S, Cardona CJ, Warnock N, Bishop MA, et al. Low Prevalence of Avian Influenza Virus in Shorebirds on the Pacific Coast of North America. Waterbirds. 2008 Dec;31(4):602–10.

Jahangir A, Ruenphet S, Ueda S, Ueno Y, Shoham D, Shindo J, et al. Avian influenza and Newcastle disease viruses from northern pintail in Japan: Isolation, characterization and inter-annual comparisons during 2006–2008. Virus Research. 2009;143(1):44–52.

Jahangir A, Watanabe Y, Chinen O, Yamazaki S, Sakai K, Okamura M, et al. Surveillance of avian influenza viruses in Northern Pintails (*Anas acuta*) Tohoku District, Japan. Avian Diseases. 2008;52:49–53.

Jonassen CM, Handeland K. Avian influenza virus screening in wild waterfowl in Norway, 2005. Avian Diseases. 2007;51(1):425–8.

Karesh WB, Uhart MM, Frere E, Gandini P, Braselton WE, Puche H, et al. Health evaluation of free-ranging rockhopper penguins (*Eudyptes chrysocomes*) in Argentina. Journal of Zoo and Wildlife Medicine. 1999 Mar;30(1):25–31.

Karunakaran D, Hinshaw V, Poss P, Newman J, Halvorson D. Influenza-A outbreaks in minnesote Turkeys due to subtype-H10N7 and possible transmission by waterfowl. Avian Diseases. 1983;27(2):357–66.

Kawamoto AHN, Mancini DAP, Pereira LE, Cianciarullo AM, Cruz AS, Dias ALF, et al. Investigation of influenza in migrating birds, the primordial reservoir and transmitters of influenza in Brazil. Braz J Microbiol. 2005 Jan-Mar;36(1):88–93.

Kawaoka Y, Chambers T, Sladen W, Webster RG. Is the gene pool of Influenza Viruses in shorebirds and gulls different from that in wild ducks. Virology. 1988;163:247–50.

Kishida N, Sakoda Y, Shiromoto M, Bai GR, Isoda N, Takada A, et al. H2N5 influenza virus isolates from terns in Australia: genetic reassortants between those of the Eurasian and American lineages. Virus Genes. 2008 Aug;37(1):16–21.

Kocan AA, Hinshaw VS, Daubney GA. Influenza a Viruses Isolated from Migrating Ducks in Oklahoma. Journal of Wildlife Diseases. 1980;16(2):281–5.

Kocan AA, Snelling J, Greiner EC. Some infectious and parasitic diseases in Oklahoma raptors. Journal of Wildlife Diseases. 1977;13(3):304–6.

Komar N, Olsen B. Avian influenza virus (H5N1) mortality surveillance. Emerging Infectious Diseases. 2008 Jul;14(7):1176–8.

Kou Z, Li YD, Yin ZH, Guo S, Wang ML, Gao XB, et al. The Survey of H5N1 Flu Virus in Wild Birds in 14 Provinces of China from 2004 to 2007. PLoS One. 2009 Sep;4(9):e6926.

Krauss S, Obert CA, Franks J, Walker D, Jones K, Seiler P, et al. Influenza in migratory birds and evidence of limited intercontinental virus exchange. PLoS Pathogens. 2007;3(11):e167.

Krauss S, Walker D, Pryor SP, Niles L, Li CH, Hinshaw VS, et al. Influenza A viruses of migrating wild aquatic birds in North America. Vector-Borne and Zoonotic Diseases. 2004 Fal;4(3):177–89.

Lai AC, McPhillips AM. Isolation of avian influenza viruses in central Oklahoma. Journal Oklahoma State Medical Association. 1999;92(12):565–7.

Langstaff IG, McKenzie JS, Stanislawek WL, Reed CEM, Poland R, Cork SC. Surveillance for highly pathogenic avian influenza in migratory shorebirds at the terminus of the East Asian-Australasian Flyway. New Zealand Veterinary Journal. 2009;57(3):160–5.

Laver WG, Webster RG. Antibodies to Human Influenza-Virus Neuraminidase (a/Asian/57 H2n2 Strain) in Sera from Australian Pelagic Birds. Bulletin of the World Health Organization. 1972;47(4):535–41.

Lebarbenchon C, Chang C-M, van der Werf S, Aubin J-T, Kayser Y, Ballesteros M, et al. Influenza A Virus in Birds during Spring Migration in the Camargue, France. Journal of Wildlife Diseases. 2007 October 1, 2007;43(4):789–93.

Lebarbenchon C, van der Werf S, Thomas F, Aubin JT, Azebi S, Cuvelier F, et al. Absence of detection of highly pathogenic H5N1 in migratory waterfowl in southern France in 2005-2006. Infection Genetics and Evolution. 2007 Sep;7(5):604–8.

Lillehaug A, Jonassen CM, Bergsjo B, Hofshagen A, Tharaldsen J, Nesse LL, et al. Screening of feral pigeon (*Colomba livia*), mallard (*Anas platyrhynchos*) and graylag goose (*Anser anser*) populations for Campylobacter spp., Salmonella spp., avian influenza virus and avian paramyxovirus. Acta Veterinaria Scandinavica. 2005;46(4):193–202.

Lindh E, Huovilainen A, Ratti O, Ek-Kommonen C, Sironen T, Huhtamo E, et al. Orthomyxo-, paramyxo- and flavivirus infections in wild waterfowl in Finland. Virology Journal. 2008 Feb;5:35.

Lipkind M, Weisman Y, Shihmanter E, Shoham D. Review of the Three-Year Studies on the Ecology of Avian Influenza Viruses in Israel. Avian Diseases. 2003;47:69–78.

L’vov DK, Easterday BC, Webster RG, Sazonov AA, Zhilina NN. [Virological and serological examination of wild birds during the spring migrations in the region of the Manych Reservoir, Rostov Province] [in Russian]. Voprosy Virusologii. 1977;Jul-Aug(4):409–14.

L’vov DK, Shchelkanov MI, Deriabin PG, Burtseva EI, Galkina IV, Grebennikova TV, et al. Highly pathogenic influenza A/H5N1 virus-caused epizooty among mute swans (*Cygnus olor*) in the lower estuary of the Volga River (November 2005) [in Russian]. Voprosy Virusologii. 2006;51(3):10–6.

L’vov DK, Shchelkanov MY, Deryabin PG, Grebennikova TV, Prilipov AG, Nepoklonov YA, et al. [Isolation of influenza A/H5N1 virus strains from poultry and wild birds in West Siberia during epizooty (July 2005) and their depositing to the state collection of viruses (August 8, 2005)] [in Russian]. Voprosy Virusologii. 2006;51(1):11–4.

L’vov DK, Yamnikova SS, Gambaryan AS, Fedyakina IT, Matrosovich MN. Isolation of influenza viruses from wild birds in the Volga River basin and in the North Caspian Region. International Congress Series. 2001;1219:251–8.

Mackenzie JS, Edwards EC, Holmes RM, Hinshaw VS. Isolation of Ortho- and Paramoxyviruses from wild birds in Western Australia, and the characterisation of novel Influenza A viruses. Australian Journal of Experimental Biology and Medical Science. 1984;62(1):89–99.

Mikami T, Izawa H, Kodama H, Onuma M, Sato A, Kobayashi S, et al. Isolation of Orthomyxovirus and paramyxovirus from migrating feral ducks in Hokkaido. Archives of Virology. 1982;74(2-3):211–7.

Mizakova A, Gronesova P, Betakova T. Monitoring of influenza viruses in waterfowl and terrestrial birds in eastern Slovakia. Acta Virologica. 2008;52(1):71–3.

Morishita TY, Aye PP, Ley EC, Harr BS. Survey of pathogens and blood parasites in free-living passerines. Avian Diseases. 1999;43(3):549–52.

Morishita TY, McFadzen ME, Mohan R, Aye PP, Brooks DL. Serologic survey of free-living nestling prairie falcons (*Falco mexicanus*) for selected pathogens. Journal of Zoo and Wildlife Medicine. 1998 Mar;29(1):18–20.

Motha J, Gibbons AM, Reed CEM. A survey for avian paramyxoviruses and influenza viruses in feral pigeons and native birds in New Zealand. New Zealand Veterinary Journal. 1997;45(5):215–6.

Müller T, Hlinak A, Freuling C, Muhle RU, Engelhardt A, Globig A, et al. Virological Monitoring of White Storks (*Ciconia ciconia*) for Avian Influenza. Avian Diseases. 2009 Dec;53(4):578–84.

Munster VJ, Baas C, Lexmond P, Waldenström J, Wallensten A, Fransson T, et al. Spatial, Temporal, and Species Variation in Prevalence of Influenza A Viruses in Wild Migratory Birds. PLoS Pathogens. 2007;3(5):e61.

Nagy A, Machova J, Hornickova J, Tomci M, Nagl I, Horyna B, et al. Highly pathogenic avian influenza virus subtype H5N1 in Mute swans in the Czech Republic. Veterinary Microbiology. 2007 Feb;120(1-2):9–16.

Nettles VF, Wood JM, Webster RG. Wildlife surveillance associated with an outbreak of lethal H5N2 avian influenza in domestic poultry. Avian Diseases. 1985;29(3):733–41.

Okazaki K, Takada A, Ito T, Imai M, Takakuwa H, Hatta M, et al. Precursor genes of future pandemic influenza viruses are perpetuated in ducks nesting in Siberia. Archives of Virology. 2000;145(5):885–93.

Oliveira JG, Belluci MSP, Vianna JSM, Mazur C, Andrade CM, Fedullo LPL, et al. [Serological survey on influenza virus in domestic and wild birds from Rio do Janeiro State, Brazil] [in Portuguese]. Arquivo Brasileiro De Medicina Veterinaria E Zootecnia. 2001;53(3):299–302.

Otsuki K, Kariya H, Matsuo K, Sugiyama S, Hoshina K, Yoshikane T, et al. Isolation of influenza-A viruses from migratory waterfowls in San-in district, Western Japan in winters of 1984-1985. Japanese Journal of Veterinary Science. 1987 Aug;49(4):721–3.

Otsuki K, Takemoto O, Fujimoto R, Kawaoka Y, Tsubokura M. Isolation of influenza-A viruses from migratory waterfowls in San-in district, Western Japan in winters of 1980-1982. Zentralblatt Fur Bakteriologie Mikrobiologie Und Hygiene Series a-Medical Microbiology Infectious Diseases Virology Parasitology. 1987;265(1-2):235–42.

Otsuki K, Takemoto O, Fujimoto R, Yamazaki K, Kubota N, Hosaki H, et al. Isolation of influenza-A viruses from migratory waterfowls in San-in district, Western Japan in winters of 1982-1983. Acta Virologica. 1987;31(5):439–42.

Otsuki K, Takemoto O, Fujimoto R, Yamazaki K, Kubota N, Hosaki H, et al. Isolation of influenza-A viruses from migratory waterfowls in San-in district, Western Japan in winters of 1983-1984. Research in Veterinary Science. 1987;43(2):177–9.

Pannwitz G, Wolf C, Harder T. Active Surveillance for Avian Influenza Virus Infection in Wild Birds by Analysis of Avian Fecal Samples from the Environment. Journal of Wildlife Diseases. 2009 Apr;45(2):512–8.

Pant GR, Selleck PW. Surveillance for avian influenza in Nepal 2004-2005. Avian Diseases. 2007;51(1):352–4.

Papanikolaou J, Koumbati-Artopiou M. Epidemiological investigation of avian influenza in regions of Northern Greece. Journal of the Hellenic Veterinary Medical Society. 2002;53(2):132–7.

Parmley EJ, Bastien N, Booth TF, Bowes V, Buck PA, Breault A, et al. Wild bird influenza survey, Canada, 2005. Emerging Infectious Diseases. 2008 Jan;14(1):84–7.

Pasick J, Handel K, Robinson J, Bowes V, Li Y, Leighton T, et al. Relationship between H5N2 avian influenza viruses isolated from wild and domestic ducks in British Columbia, Canada. Avian Diseases. 2007;51:429–31.

Pawar S, Pande S, Jamgaonkar A, Koratkar S, Pal B, Raut S, et al. Avian influenza surveillance in wild migratory, resident, domestic birds and in poultry in Maharashtra and Manipur, India, during avian migratory season 2006-07. Current Science. 2009 Aug;97(4):550–4.

Pearce JM, Ramey AM, Flint PL, Koehler AV, Fleskes JP, Franson JC, et al. Avian influenza at both ends of a migratory flyway: characterizing viral genomic diversity to optimize surveillance plans for North America. Evolutionary Applications. 2009 Nov;2(4):457–68.

Pereda AJ, Uhart M, Perez AA, Zaccagnini ME, La Sala L, Decarre J, et al. Avian influenza virus isolated in wild waterfowl in Argentina: Evidence of a potentially unique phylogenetic lineage in South America. Virology. 2008 Sep;378(2):363–70.

Peroulis I, O'Riley K. Detection of avian paramyxoviruses and influenza viruses amongst wild bird populations in Victoria. Australian Veterinary Journal. 2004 Jan-Feb;82(1-2):79–82.

Peterson AT, Bush SE, Spackman E, Swayne DE, Ip HS. Influenza A virus infections in land birds, People's Republic of China. Emerging Infectious Diseases. 2008 Oct;14(10):1644–6.

Peterson MJ, Aguirre R, Ferro PJ, Jones DA, Lawyer TA, Peterson MN, et al. Infectious disease survey of Rio Grande wild turkeys in the Edwards Plateau of Texas. Journal of Wildlife Diseases. 2002;38(4):826–33.

Peterson MJ, Ferro PJ, Peterson MN, Sullivan RM, Toole BE, Silvy NJ. Infectious disease survey of lesser prairie chickens in north Texas. Journal of Wildlife Diseases. 2002;38(4):834–9.

Pfitzer S, Verwoerd DJ, Gerdes GH, Labuschagne AE, Erasmus A, Manvell RJ, et al. Newcastle disease and avian influenza A virus in wild waterfowl in South Africa. Avian Diseases. 2000 Jul-Sep;44(3):655–60.

Rabl S, Rinder M, Neubauer-Juric A, Bogner KH, Korbel R, Buttner M. [Surveillance of wild birds for Avian Influenza A Virus (AIV) in Bavaria in the years 2007 and 2008] [in German]. Berliner Und Munchener Tierarztliche Wochenschrift. 2009 Nov-Dec;122(11-12):486–93.

Račnik J, Slavec B, Trilar T, Zadravec M, Dovč A, Krapež U, et al. Evidence of avian influenza virus and paramyxovirus subtype 2 in wild-living passerine birds in Slovenia. European Journal of Wildlife Research. 2008;54:529–32.

Raleigh PJ, Flynn O, O'Connor M, O'Donovan T, Purcell B, De Burca M, et al. Avian influenza viruses detected by surveillance of waterfowl in Ireland during 2003-2007. Epidemiology and Infection. 2009 Apr;137(4):464–72.

Razumova IV, Shchelkanov MI, Zolotykh SI, Durymanova AA, Ternovoi VA, Beklemishev AB, et al. [The 2003 results of monitoring of influenza A virus in the populations of wild birds in the south of Western Siberia] [in Russian]. Voprosy Virusologii. 2006;51(3):32–7.

Romváry J, Mészáros J, Tanyi J, Rózsa J, Fábián L. Influenza infectedness of captured and shot wild birds on north-eastern and south-eastern parts of Hungary. Acta Veterinaria Academiae Scientiarum Hungaricae. 1976;26(3):363–8.

Rosenberger JK, Krauss WC, Slemons RD. Isolation of Newcastle-disease and Type-A Influenz a Viruses from migratory waterfowl in Atlantic Flyway. Avian Diseases. 1974;18(4):610–3.

Runstadler JA, Happ GM, Slemons RD, Sheng Z-M, Gundlach N, Petrula M, et al. Using RRT-PCR analysis and virus isolation to determine the prevalence of avian influenza virus infections in ducks at Minto Flats State Game Refuge, Alaska, during August 2005. Archives of Virology. 2007.

Rutz C, Dalessi S, Baumer A, Kestenholz M, Engels M, Hoop R. Avian influenza: Wildbird monitoring in Switzerland between 2003–2006 [in German]. Schweizer Archiv Fur Tierheilkunde. 2007 Nov;149(11):501–9.

Schnebel B, Dierschke V, Rautenschlein S, Ryll M, Neumann U. Investigations on infection status with H5 and H7 avian influenza virus in short-distance and long-distance migrant birds in 2001. Avian Diseases. 2007;51(1):432–3.

Sekler M, Ruzica A, Krnjaic D, Palic T, Milic N, Tanja J, et al. Examination of presence os specific antibodies against avian influenza virus in some species of wild birds. Acta Veterinaria-Beograd. 2009;59(4):381–402.

Sharp GB, Kawaoka Y, Wright SM, Turner B, Hinshaw V, Webster RG. Wild ducks are the reservoir for only a limited number of influenza A subtypes. Epidemiology and Infection. 1993;110:161–76.

Sharshov KA, Zolotykh SI, Fedorov EG, Ivanov IV, Druziaka AV, Shestopalov AM, et al. Surveillance for avian influenza virus in synanthropic birds during epizootic and postepizootic periods on the south of west Siberia [in Russian]. Zhurnal Mikrobiologii Epidemiologii i Immunobiologii. 2007(4):53–6.

Shchelkanov MY, Ananyev VY, Lvov DN, Kireyev DY, Guryev YL, Akanina DS, et al. Complex environmental and virological monitoring in the Primorye Territory in 2003–2006 [in Russian]. Voprosy Virusologii. 2007;52(5):37–48.

Shengqing Y, Shinya K, Otsuki K, Ito H, Ito T. Isolation of myxoviruses from migratory waterfowls in San-in district, western Japan in winters of 1997-2000. Journal of Veterinary Medical Science. 2002;64(11):1049–52.

Shestopalov AM, Solotych SI, Shchelkanov MY, Razumova YV, Alekseev AY, Durymanov AG, et al. [Results of two-year-old inspection of wild birds in territory of the Western Mongolia on the presence infuenza virus] [in Russian]. Zhurnal Mikrobiologii Epidemiologii i Immunobiologii. 2006(5):55–9.

Simulundu E, Mweene AS, Tomabechi D, Hang'ombe BM, Ishii A, Suzuki Y, et al. Characterization of H3N6 avian influenza virus isolated from a wild white pelican in Zambia. Archives of Virology. 2009 Sep;154(9):1517–22.

Sinnecker H, Sinnecker R, Zilske E, Koehler D. Detection of influenza A viruses and influenza epidemics in wild pelagic birds by sentinels and population studies. Zentralblatt Fur Bakteriologie Mikrobiologie Und Hygiene Series a-Medical Microbiology Infectious Diseases Virology Parasitology. 1982;253(3):297–304.

Sinnecker R, Sinnecker H, ZIlske E, Kohler D. Surveillance of pelagic birds for influenza A viruses. Acta Virologica. 1983;27:75–9.

Slemons RD, Hansen WR, Converse KA, Senne DA. Type A influenza virus surveillance in free-flying, nonmigratory ducks residing on the eastern shore of Maryland. Avian Diseases. 2003;47:1107–10.

Slemons RD, Johnson DC, Osborn JS, Hayes F. Type-A Influenza-Viruses Isolated from Wild Free-Flying Ducks in California. Avian Diseases. 1974;18(1):119–24.

Slemons RD, Shieldcastle MC, Heyman LD, Bednarik KE, Senne DA. Type-A Influenza-Viruses in Waterfowl in Ohio and Implications for Domestic Turkeys. Avian Diseases. 1991 Jan-Mar;35(1):165–73.

Slepuskin AN, Pysina TV, Gonsovsky FK, Sazonov AA, Isacenko VA, Sokolova NN, et al. Haemagglutination-inhibiting activity to type A influenza viruses in the sera of wild birds from the far east of the USSR. Bulletin of the World Health Organization. 1972;47(4):527–30.

Smietanka K, Minta Z, Domanska-Blicharz K, Tomczyk G, Wijaszka T. Avian influenza H5N1 outbreak in a flock of Mute swans in the city of Torun, Poland, in 2006. Bulletin of the Veterinary Institute in Pulawy. 2008;52(4):491–5.

Smietanka K, Minta Z, Tomczyk G, Domanska-Blicharz K, Bartnicka B, Szewczyk B, et al. Prevalence of avian influenza virus infections in poultry and wild birds. Medycyna Weterynaryjna. 2005 Jun;61(6):676–9.

Smith GJD, Vijaykrishna D, Ellis TM, Dyrting KC, Leung YHC, Bahl J, et al. Characterization of Avian Influenza Viruses A (H5N1) from Wild Birds, Hong Kong, 2004-2008. Emerging Infectious Diseases. 2009 Mar;15(3):402–7.

Smith KM, Karesh WB, Majluf P, Paredes R, Zavalaga C, Reul AH, et al. Health Evaluation of Free-Ranging Humboldt Penguins (*Spheniscus humboldti*) in Peru. Avian Diseases. 2008 Mar;52(1):130–5.

Smitka CW, Maassab HF. Ortho-myxoviruses and paramyxoviruses in the migratory waterfowl of Michigan. Journal of Wildlife Diseases. 1981;17(1):147–51.

Song MS, Oh TK, Moon HJ, Yoo DW, Lee EH, Lee JS, et al. Ecology of H3 avian influenza viruses in Korea and assessment of their pathogenic potentials. Journal of General Virology. 2008 Apr;89:949–57.

Spackman E, McCracken KG, Winker K, Swayne DE. An avian influenza virus from waterfowl in South America contains genes from North American avian and equine lineages. Avian Diseases. 2007;51(1):273–4.

Spackman E, Swayne DE, Gilbert M, Joly DO, Karesh WB, Suarez DL, et al. Characterization of low pathogenicity avian influenza viruses isolated from wild birds in Mongolia 2005 through 2007. Virology Journal. 2009 Nov;6:8.

Stallknecht DE, Shane SM, Zwank PJ, Senne DA, Kearney MT. Avian Influenza-Viruses from Migratory and Resident Ducks of Coastal Louisiana. Avian Diseases. 1990 Apr-Jun;34(2):398-405.

Stanislawek WL. Avian influenza survey of New Zealand wild ducks. Surveillance. 1990;17(2):13–4.

Stanislawek WL. Survey of wild ducks for evidence of avian influenza viruses, 1989 and 1990. Surveillance. 1992;19(1):21–2.

Stanislawek WL, Wilks CR, Meers J, Horner GW, Alexander DJ, Manvell RJ, et al. Avian paramyxoviruses and influenza viruses isolated from mallard ducks (*Anas platyrhynchos*) in New Zealand. Archives of Virology. 2002;147(7):1287–302.

Stone EG, Montiel-Parra G, Perez TM. A survey of selected parasitic and viral pathogens in four species of Mexican parrots, *Amazona autumnalis*, *Amazona oratrix*, *Amazona viridigenalis*, and *Rhynchopsitta pachyrhyncha*. Journal of Zoo and Wildlife Medicine. 2005;36(2):245–9.

Stoops AC, Barbara KA, Indrawan M, Ibrahim IN, Petrus WB, Wijaya S, et al. H5N1 Surveillance in Migratory Birds in Java, Indonesia. Vector-Borne and Zoonotic Diseases. 2009 Dec;9(6):695–702.

Süss J, Schafer J, Sinnecker H, Webster RG. Influenza virus subtypes in aquatic birds of eastern Germany. Archives of Virology. 1994;135:101–14.

Tanaka T, Tanoue G, Yamasaki M, Takashima I, Sakoda Y, Ochiai K, et al. Chemical deicer poisoning was suspected as a cause of the 2005-2006 wintertime mortality of small wild birds in Hokkaido. Journal of Veterinary Medical Science. 2008 Jun;70(6):607–10.

Terregino C, De Nardi R, Guberti V, Scremin M, Raffini E, Martin AM, et al. Active surveillance for avian influenza viruses in wild birds and backyard flocks in Northern Italy during 2004 to 2006. Avian Pathology. 2007;36(4):337–44.

Toro H, Pavez EF, Gough RE, Montes G, Kaleta EF. Serum chemistry and antibody status to some avian pathogens of free-living and captive condors (*Vultur gryphus*) of central Chile. Avian Pathology. 1997;26(2):339–45.

Travis EK, Vargas FH, Merkel J, Gottdenker N, Miller RE, Parker PG. Hematology, serum chemistry, and serology of Galapagos penguins (Spheniscus mendiculus) in the Galapagos Islands, Ecuador. Journal of Wildlife Diseases. 2006 Jul;42(3):625–32.

Travis EK, Vargas FH, Merkel J, Gottdenker N, Miller RE, Parker PG. Hematology, plasma chemistry, and serology of the flightless cormorant (*Phalacrocorax harrisi*) in the Galapagos Islands, Ecuador. Journal of Wildlife Diseases. 2006 Jan;42(1):133–41.

Tsubokura M, Otsuki K, Kawaoka Y, Yanagawa R. Isolation of influenza A viruses from migratory waterfowls in San-in District, Western Japan in 1979-1980. Zentralblatt Fur Bakteriologie

Mikrobiologie Und Hygiene Serie B-Umwelthygiene Krankenhaushygiene Arbeitshygiene Praventive Medizin. 1981;173(6):494–500.

Uhart M, Aprile G, Beldomenico R, Solis G, Marull C, Beade M, et al. Evaluation of the health of free-ranging greater rheas (*Rhea americana*) in Argentina. Veterinary Record. 2006 Mar;158(9):297–303.

Uhart MM, Quintana F, Karesh WB, Braselton WE. Hematology, plasma biochemistry, and serosurvey for selected infectious agents in southern giant petrels from Patagonia, Argentina. Journal of Wildlife Diseases. 2003 Apr;39(2):359–65.

Wahlgren J, Waldenstrom J, Sahlin S, Haemig PD, Fouchier RAM, Osterhaus A, et al. Gene Segment Reassortment Between American and Asian Lineages of Avian Influenza Virus from Waterfowl in the Beringia Area. Vector-Borne and Zoonotic Diseases. 2008 Dec;8(6):783–90.

Wallensten A, Munster VJ, Latorre-Margalef N, Brytting M, Elmberg J, Fouchier RAM, et al. Surveillance of influenza A virus in migratory watefowl in northern Europe. Emerging Infectious Diseases. 2007;13(3):404–11.

Wallensten A, Munster VJ, Osterhaus A, Waldenstrom J, Bonnedahl J. Mounting evidence for the presence of influenza A virus in the avifauna of the Antarctic region. Antarctic Science. 2006;18:353–6.

Webster RG, Morita M, Pridgen C, Tumova B. Ortho- and Paramyxoviruses from Migrating Feral Ducks: Characterization of a New Group of Influenza A Viruses. Journal of General Virology. 1976;32:217–25.

Winker K, McCracken KG, Gibson DD, Pruett CL, Meier R, Huettmann F, et al. Movements of birds and avian influenza from Asia into Alaska. Emerging Infectious Diseases. 2007;13(4):547–52.

Winkler WG, Trainer DO, Easterday BC. Influenza in Canada geese. Bulletin of the World Health Organization. 1972;47(4):507–13.

Yamane N, Odagiri T, Arikawa J, Ishida N. Isolation and characterization of influenza A viruses from wild ducks in northern Japan: appearance of HSW1 antigens in the Japanese duck population. Acta Virologica. 1979;23(5):375–84.

Zakstel'skaja LJ, Isacenko VA, Osidze NG, Timofeeva CC, Slepushkin AN, Sokolova NN. Some observations on the circulation of influenza viruses in domestic and wild birds. Bulletin of the World Health Organization. 1972;47(4):497–501.

Zarkov IS, Bochev I, Manvell R, Shell W. Isolation of avian influenza virus in Bulgaria. Veterinary Record. 2006 Jan;158(3):106–7.

Zeng X, Hua Y, Li X, Zhang Z. Monitoring Influenza A virus and Newcastle disease virus in migratory waterfowls in Sanjiang natural Reserve of Heilongjiang Province [in Chinese]. Weishengwu Xuebao. 2008;48(10):1403–7.

Estimating Minimum Detectable Prevalence

To determine probability of detecting at least one infected individual, let p be the prevalence of infection in a very large population (in which infected individuals are homogenously distributed). A randomly chosen individual from this population therefore has a probability of p of being infected, but also a probability equal to (1-p) of not being infected. If we sample n individuals from this population at random, the probability that none of them are infected is $(1-p)^n$. Thus the probability of finding at least one infected individual ($P_{x>0}$) is then:

$$P_{x>0} = 1 - (1 - p)^n \qquad (1)$$

Rearranging equation 1, we can calculate how many individuals to sample (n) to be ($P_{x>0}$) confident of detecting at least one infected individual when prevalence is above some pre-defined threshold (p_{max}):

$$n = \frac{\log(1 - P_{x>0})}{\log(1 - p_{max})} \qquad (2)$$

While prevalence is rarely known before initiating a survey, a conservative limit of detection should be used; a nominal prevalence of 0.5% (i.e. n=597) has been suggested, indicating that at least 600 samples are required to achieve 95% confidence of disease freedom.

The maximum prevalence (p_{max}) of infection that could have been in the population is also calculable if all n individuals were negative:

$$p_{max} = 1 - (1 - P_{x>0})^{\frac{1}{n}} \qquad (3)$$

For example, if 300 individual birds were tested but no infection was detected, the study can be 95% confident that prevalence is less than 1%.
